# MateR: a novel genomic mating framework

**DOI:** 10.1093/genetics/iyag013

**Published:** 2026-01-19

**Authors:** Javier Fernández-González, Seifelden M Metwally, Julio Isidro y Sánchez

**Affiliations:** Centro de Biotecnologia y Genómica de Plantas (CBGP, UPM-INIA), Universidad Politécnica de Madrid (UPM) - Instituto Nacional de Investigación y Tecnologia Agraria y Alimentaria (INIA), Campus de Montegancedo-UPM, Pozuelo de Alarcón, Madrid 28223, Spain; Centro de Biotecnologia y Genómica de Plantas (CBGP, UPM-INIA), Universidad Politécnica de Madrid (UPM) - Instituto Nacional de Investigación y Tecnologia Agraria y Alimentaria (INIA), Campus de Montegancedo-UPM, Pozuelo de Alarcón, Madrid 28223, Spain; Centro de Biotecnologia y Genómica de Plantas (CBGP, UPM-INIA), Universidad Politécnica de Madrid (UPM) - Instituto Nacional de Investigación y Tecnologia Agraria y Alimentaria (INIA), Campus de Montegancedo-UPM, Pozuelo de Alarcón, Madrid 28223, Spain

**Keywords:** genomic mating, optimal mate allocation, optimal contribution selection, genomic prediction, usefulness criterion, inbreeding control

## Abstract

Genomic mating uses genome-wide information to design crosses that maximize genetic gain while managing diversity. Expected gain is often predicted through the usefulness criterion, which depends on family means and variances. However, existing equations mix incompatible parameterizations when considering dominance effects. Furthermore, diversity control is often tuned with metrics that lack a direct link to the loss of additive variation and long-term gain. We derived equations that compute family mean and within-family variance consistently under breeding and genotypic parameterizations by computing locus-specific values using genotypic frequencies and propagating them to the entire genome through linkage disequilibrium covariances. We also developed a diversity metric that estimates the proportion of additive standard deviation lost and integrated both advances into the MateR software. We evaluated performance in simulated diploid and autotetraploid crop populations across multiple breeding schemes and against existing tools. The new equations predicted family means and variances with near-perfect accuracy when true QTL effects were known. With estimated marker effects, correlations were roughly 0.55–0.90 for family mean and about 0.25 for within-family standard deviation. The diversity metric matched the expected loss of additive standard deviation under random sampling and tracked loss of genic variance under selection. This framework unifies prediction of cross usefulness under dominance and supplies an interpretable diversity control directly tied to long-term gain. Implemented in MateR, it applies to diploids and autopolyploids and accommodates common breeding program constraints, including hybrid schemes and testers.

## Introduction

Breeding for quantitative traits relies on crossing elite parents to generate new diversity and select superior genotypes. Optimizing a mating plan requires predicting the outcome of every possible cross, which has been made possible by genomic prediction (GP) (Meuwissen et al. 2001). This optimization, often termed genomic mating or optimal mate allocation (Kinghorn 2011; Akdemir and Isidro y Sánchez 2016; Gorjanc and Hickey 2018), aims to maximize genetic gain as described by the breeder’s equation:


(1) $$R_{t}=\frac{ir\sigma_{a}}{L}$$

Where $R_{t}$ is the response to selection per unit of time, *i* is selection intensity, *r* is prediction accuracy, $\sigma_{a}$ is the additive genetic standard deviation, and *L* is the generation interval (Lynch and Walsh 1998). In genomic mating, *r* and *L* are typically held fixed, whereas the mating plan can alter *i* and $\sigma_{a}$. Prioritizing only the best parents inflates *i* in the short term but rapidly depletes $\sigma_{a}$ across cycles. Thus, early implementations (Kinghorn 2011; Gorjanc and Hickey 2018) balanced two objectives, maximizing the average merit of selected parents (strongly related to *i*) and maintaining genomic diversity to limit the loss of $\sigma_{a}$. This is a relatively simple and effective strategy (Endelman 2025) that nonetheless partially overlooks parental complementarity at the cross level.

To overcome this limitation, it is important to consider within-family variance, which affects the chance of sampling exceptional progeny. To account for this, the *usefulness criterion* (Schnell and Utz 1975; Lehermeier et al. 2017b; Zhong and Jannink 2007) was developed to integrate family mean and variance. Historically, it could only be measured by phenotyping the progeny, which would defeat the purpose of optimizing the mating plan. In contrast, nowadays GP allows for the prediction of both quantities for any pair of genotyped parents (Lehermeier et al. 2017b), enabling refined genomic mating. Akdemir and Isidro y Sánchez (2016) incorporated within-family variance but neglected dominance. Recent work extended usefulness to incorporate dominance effects (Wolfe et al. 2021; Peixoto et al. 2025), but they unintentionally mixed distinct GP parameterizations, which compromises predictions.

The commonly used *breeding parameterization* (Vitezica et al. 2013) decomposes the total genotypic value into breeding values and dominance deviations in the absence of epistasis. The breeding value of a genotype is its expected progeny mean when mated at random to a reference population and includes all additive effects plus the expected dominance. The dominance deviations are simply the difference between the total genotypic value and the breeding values. Critically, breeding values and dominance deviations are independent, which is a key assumption made by Wolfe et al. (2021) and Peixoto et al. (2025) when computing family variance. In contrast, their computation of family mean follows Falconer (1989) (see Equation (2)). This approach assumes the *genotypic parameterization*, which decomposes the total genotypic value into strictly additive and dominance components that are not mutually independent (Vitezica et al. 2013). Consequently, adopting the breeding parameterization miscomputes the mean, while adopting the genotypic parameterization violates the variance assumptions. Our first objective is therefore to derive equations for family mean and variance that are valid under both parameterizations.

Our second objective addresses diversity control. Genomic mating balances usefulness against the expected depletion of $\sigma_{a}$ via a tunable user-defined hyperparameter. We argue that an interpretable diversity metric simplifies this tuning process. Common approaches rely on parental kinship (Akdemir and Isidro y Sánchez 2016; Peixoto et al. 2025) or kinship-based angles (Kinghorn 2011; Gorjanc and Hickey 2018). Although effective, these metrics are difficult to interpret and tune because their link to specific levels of $\sigma_{a}$ depletion and long-term gain is not straightforward. The classical inbreeding rate (Toro and Perez-Enciso 1990; Wray and Thompson 1990; Wray and Goddard 1994; Meuwissen 1997; Grundy et al. 1998; Endelman 2025) is interpretable but difficult to estimate robustly from markers without pedigrees that distinguish identity by descent (IBD) from identity by state (IBS) (Wang 2014; Endelman 2024). This issue could be solved by employing runs of homozygosity (ROH) (Peripolli et al. 2017), but to our knowledge, progeny ROH can only be estimated by simulating offspring genomes, which is very computationally intensive. Furthermore, ROH does not inherently control group coancestry. Families with low expected ROH-based inbreeding could be strongly related to each other, which would be detrimental to long-term genetic variability. Finally, regardless of how it is computed, the inbreeding rate does not directly quantify the expected reduction in $\sigma_{a}$ per cycle. It is known that inbreeding is related to variance through $\sigma_{a}^{2}=\sigma_{a_{0}}^{2}(1-F)$, where $\sigma_{a}^{2}$ is the variance of the current population, $\sigma_{a_{0}}^{2}$ is the variance in the base population, and *F* is the inbreeding coefficient. However, this equation applies only to an idealized population under Hardy-Weinberg equilibrium (Falconer 1989), limiting its applicability in practical breeding contexts. Therefore, here we aim to develop a diversity metric that is directly interpretable as the anticipated reduction in $\sigma_{a}$ for a given mating plan.

Finally, our third objective is to implement these developments in a new R package called MateR, delivering genomic mating with (i) parameterization-consistent usefulness under dominance and (ii) an interpretable handle on diversity loss. We emphasize versatility, supporting autopolyploid species, clonal, self-pollinated, and hybrid crops; as well as animal breeding.

## Mathematical development

For clarity, we present derivations under the *genotypic parameterization* in this section, but they also hold under the *breeding parameterization* provided the corresponding marker scores and effects are used.

### Family mean

The family mean is often computed as the mid-parent value (valid only under additivity) or using Equation 14.6 in Falconer (1989):


(2) $$\mu_{F_{1}}=a\;(p-q-y)+d\;[2pq+y(p-q)],$$

which gives the expected genotypic value of the $F_{1}$ from two distinct parental populations. This expression is restricted to $F_{1}$ and the genotypic parameterization, and in our tests , it did not reproduce the correct expectations (Supplementary File 1). We therefore propose a more general and accurate method based on genotypic frequencies.

Let *F* denote the family generated by crossing parents $P_{k}$ and $P_{l}$. Consider locus *j* with alleles *A* and *a*, and additive and dominance effects $a_{j}$ and $d_{j}$. In diploids, the additive marker score M[i,j]∈{0,1,2} for individual *i* is encoded as the count of allele *a* (AA=0, Aa=1, aa=2), and the dominance score W[i,j]∈{0,1} is a heterozygosity indicator (AA=0, Aa=1, aa=0). Let $PAA_{F,j}$, $PAa_{F,j}$, and $Paa_{F,j}$ be the genotypic frequencies in *F* at locus *j* (summing to 1). The locus-specific family mean can be simply calculated by applying the definition of the arithmetic mean:


(3) $$\mu_{F,j}=Paa_{F,j}(2a_{j}+0d_{j})+PAa_{F,j}(1a_{j}+1d_{j})+PAA_{F,j}(0a_{j}+0d_{j})$$

This overcomes the limitations of Equation (2) by generalizing to any parameterization, filial generation and degree of ploidy (Appendix A).

Equation (3) refers to a single locus, but it can be easily extended to the entire genome. The total genotypic value ($g_{i}$) of an individual *i* is computed by summing the additive and dominance effects across all $n_{j}$ loci:


$$g_{i}=\sum_{\;j=1}^{n_{j}}M[i,j]a_{j}+W[i,j]d_{j}$$

Accordingly, the expected genotypic value of a family *F* is:


(4) $$\begin{array}{rl} E(g_{F}) & =E\;\left(\sum_{\;j=1}^{n_{j}}M[F,j]a_{j}+W[F,j]d_{j}\right)\\ & =\sum_{\;j=1}^{n_{j}}E\;\left(M[F,j]a_{j}+W[F,j]d_{j}\right)=\sum_{\;j=1}^{n_{j}}\mu_{F,j} \end{array}$$

### Within-family variance

Using the same notation as above and the formula for population variance, the per-locus within-family variance for family *F* at locus *j* is


(5) $$\begin{array}{rl} \sigma_{F,j}^{2} & =Paa_{F,j}(2a_{j}+0d_{j}-\mu_{F,j}{)}^{2}+PAa_{F,j}(1a_{j}+1d_{j}-\mu_{F,j}{)}^{2}\\ & \;+PAA_{F,j}(0a_{j}+0d_{j}-\mu_{F,j}{)}^{2} \end{array}$$

where $\sigma_{F,j}^{2}$ is the within-family variance for locus *j* and $\mu_{F,j}$ is calculated from Equation (3). Extensions to autopolyploids follow by replacing the diploid genotype encodings with their polysomic counterparts, see Appendix A.

Across the genome, the variance of the sum of locus contributions includes both locus variances and between-loci covariances:


$$var(g_{F})=var\left(\sum_{\;j=1}^{n_{j}}g_{F,j}\right)=\sum_{\;j=1}^{n_{j}}\sigma_{F,j}^{2}+2\sum_{\;j_{1}\neq j_{2}}cov(g_{F,j_{1}},g_{F,j_{2}})$$

In the literature, this has been computed as (Lehermeier et al. 2017a, 2017b; Wolfe et al. 2021; Peixoto et al. 2025):


(6) $$var(g_{F})=a^{T}\Sigma_{a}a+d^{T}\Sigma_{d}d$$

where a and d are vectors of additive and dominance marker effects, and $\Sigma_{a}$ and $\Sigma_{d}$ are the covariance matrices between the additive and dominance marker scores (doses). This works fine for the breeding parameterization, which assumes that additive and dominance effects are independent. However, for the genotypic parameterization, we would need to consider their correlation. Therefore, we propose an alternative formulation where we instead express the genome-wide variance via single-locus standard deviations and their correlations.


(7) $$var(g_{F})={\sqrt{V_{F}}}^{T}C_{F}\sqrt{V_{F}}$$

Where $V_{F}=\{\sigma_{F,1}^{2},\sigma_{F,2}^{2},\ldots ,\sigma_{F,n_{j}}^{2}\}$ are locus-specific variances from Equation (5) and $C_{F}$ is the correlation matrix between loci, i.e. it reflects the linkage disequilibrium (LD). Importantly, $C_{F}$ is the correlation of the genotypic effects of the loci, not just of the marker scores. This formulation implicitly accommodates any correlation between additive and dominance effects present under the genotypic parameterization. Computing $C_{F}$ is complicated, and we have developed three options, ranging from very simplistic to very realistic:

(1) **Independent loci.** Assume $C_{F}=I$, where *I* is an identity matrix with the needed dimensions. This results in $var(g_{F})={\sqrt{V_{F}}}^{T}I\sqrt{V_{F}}=\sum_{\;j=1}^{n_{j}}\sigma_{F,j}^{2}$ and is equivalent to the approach in Akdemir and Isidro y Sánchez (2016).
(2) **Parental-LD propagation.**  $C_{F}$ can be computed as:
  (8) $$C_{F}[i,j]=\;\frac{\Sigma_{F}[i,j]}{\sqrt{\Sigma_{F}[i,i]\cdot \Sigma_{F}[j,j]}}$$

Where $\Sigma_{F}$ refers to the covariance matrix across loci for the genotypic values in family *F*. $\Sigma_{F}$ can be calculated as:


(9) $$\Sigma_{F}=f(\Sigma_{k},\Sigma_{l},c)$$

Where $\Sigma_{k}$ and $\Sigma_{l}$ denote the covariance matrices of the parental lines, and *c* is the matrix of recombination frequencies. The function in Equation (9) depends on the breeding scenario. For example, when the parents are not fully homozygous and the *F* family corresponds to the F1 generation,


(10) $$\Sigma_{F}=\frac{1}{2}\;(1-2c)⊙\Sigma_{k}+\frac{1}{2}\;(1-2c)⊙\Sigma_{l},$$

where ⊙ denotes the Hadamard (elementwise) product. See Lehermeier et al. (2017b) for expressions of $\Sigma_{F}$ for doubled haploids and for any number of selfing generations.

The last step is to compute $\Sigma_{k}$ and $\Sigma_{l}$ to evaluate the variance. The same procedure applies to both parents; for brevity, we illustrate it for parent $P_{k}$:

- Define a matrix of genotypic effects for the population from which the parent $P_{k}$ was taken:
  $$M^{*}[i,j]=M[i,j]a_{j}+W[i,j]d_{j}$$
- Center columns to obtain $Z^{*}$.
- Compute:
  (11) $$\Sigma_{k}=\;\frac{(Z^{*}{)}^{T}Z^{*}}{n}$$

Where *n* is the number of individuals in the population. In summary, within-family variance can be calculated by applying equations (5), (11), (9), (8), and (7) in that order.

(3) **Compute LD for each individual parent:**

In the previous scenario, we assumed that LD in each parent equals LD in its source population. A more realistic approach is to compute LD for specific parents, which requires phased genotypes (trivial for fully homozygous lines but rarely available otherwise).

Let $H_{k}$ be the $\phi \times n_{j}$ haplotype matrix for parent $P_{k}$, where *ϕ* is the degree of ploidy, rows represent chromosomes and columns represent markers. Each cell is coded as 0 (reference allele) or 1 (alternative allele).

We compute allele frequencies in the family as the average from both parents:


$$p_{j}=\;\frac{\frac{1}{\phi }\sum_{r=1}^{\phi }H_{k}[r,j]+\frac{1}{\phi }\sum_{r=1}^{\phi }H_{l}[r,j]}{2}$$

To compute the covariance matrix of additive values across loci, we define the following:

- Centered matrix of phased additive effects:
  $$H_{k}^{*}[r,j]=(H_{k}[r,j]-p_{j})\cdot a_{j}$$
- Additive covariance matrix:
  $$\Sigma_{a,k}=\;\frac{(H_{k}^{*}{)}^{T}H_{k}^{*}}{\phi }$$

It is important to note that, in this scenario, the covariance matrix $\Sigma_{a,k}$ does not reflect any dominance. The reason for this is that the phased information in $H_{k}$ is purely additive, and there is no way to get phased dominance marker scores, as the dominance in a locus is not assigned to a specific chromosome, but it is rather the outcome of the interaction among all of them. Next, it is also important to highlight that $p_{j}$ has to be the allele frequency in the family. The other possibility would be setting $p_{j}$ as the allele frequency in parent $P_{k}$ (Wolfe et al. 2021), but this would cause all homozygous loci to become monomorphic. As a result, their variance and covariances would become zero, which is not realistic.

Next, we can find the additive covariance between loci in the family from $\Sigma_{a,k}$, $\Sigma_{a,l}$ and *c* using Equation (9). It can be subsequently converted into a correlation matrix $C_{a,F}$ using Equation (8).

Finally, we need to include dominance. To that end, the first step is finding the additive and dominance variances within each locus ($\sigma_{a,F,j}^{2}=var(M[F,j]a_{j})$ and $\sigma_{d,F,j}^{2}=var(W[F,j]d_{j})$). They can be obtained using Equation (5) for total variance and setting $d_{j}=0$ or $a_{j}=0$ to isolate additive or dominance variance respectively. Finding them allows us to calculate the correlation between additive and dominance effects. For any locus *j* in family *F*, the genotypic variance is:


$$\sigma_{F,j}^{2}=var(M[F,j]a_{j}+W[F,j]d_{j})$$

Expanding this:


$$\sigma_{F,j}^{2}=\sigma_{a,F,j}^{2}+\sigma_{d,F,j}^{2}+2\;cov(M[F,j]a_{j},\;W[F,j]d_{j})$$

Solving for the covariance and correlation gives:


$$\begin{array}{rl} cov(M[F,j]a_{j},\;W[F,j]d_{j}) & =\;\frac{\sigma_{F,j}^{2}-\sigma_{a,F,j}^{2}-\sigma_{d,F,j}^{2}}{2}\\ \rho_{F,j} & =\;\frac{cov(M[F,j]a_{j},\;W[F,j]d_{j})}{\sigma_{a,F,j}\cdot \sigma_{d,F,j}} \end{array}$$

**Fig. 1.**
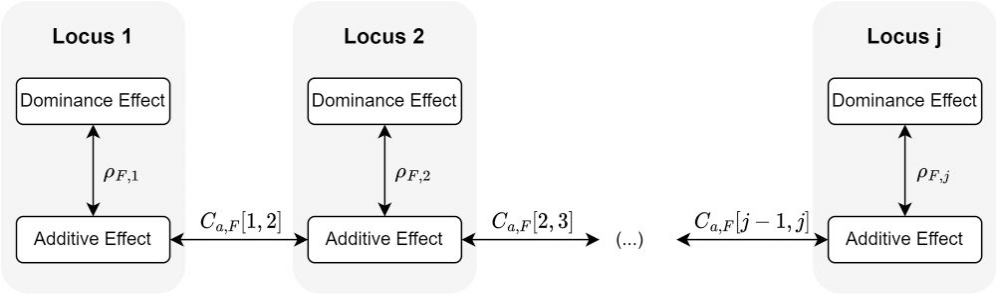
Scheme of the path analysis for the additive and dominance effects in each locus for family *F*.

These expressions allow us to obtain the within-family genotypic variance using path analysis Wright (1934). For instance, following Fig. 1, we can get the correlation between the dominance effects in loci 1 and 2 as $\rho_{F,1}C_{a,F}[1,2]\rho_{F,2}$.

Using Fig. 1, and the bilinearity of covariance, we can compute the total within-family genetic variance by summing all additive and dominance covariance components:


(12) $$\begin{array}{rl} var(g_{F}) & =var\left(\sum_{\;j=1}^{n_{j}}M[F,j]a_{j}+W[F,j]d_{j}\right)\\ & =\sum_{\;j_{1}=1}^{n_{j}}\sum_{\;j_{2}=1}^{n_{j}}cov[(M[F,j_{1}]a_{\;j_{1}}+W[F,j_{1}]d_{\;j_{1}}),\\ & \;\left(M[F,j_{2}]a_{\;j_{2}}+W[F,j_{2}]d_{\;j_{2}})\right]\\ & =\sum_{\;j_{1}=1}^{n_{j}}\sum_{\;j_{2}=1}^{n_{j}}\left[cov(M[F,j_{1}]a_{\;j_{1}},M[F,j_{2}]a_{\;j_{2}}\right)\\ & \;+cov(M[F,j_{1}]a_{\;j_{1}},W[F,j_{2}]d_{\;j_{2}})\\ & \;+cov(W[F,j_{1}]d_{\;j_{1}},M[F,j_{2}]a_{\;j_{2}})\\ & \;+cov(W[F,j_{1}]d_{\;j_{1}},W[F,j_{2}]d_{\;j_{2}})] \end{array}$$

Each of the terms above can be computed using $C_{a,F}$ and $\rho_{F,j}$ as follows:

**Additive** × **additive covariances:**


$$cov(M[F,j_{1}]a_{\;j_{1}},M[F,j_{2}]a_{\;j_{2}})=\sigma_{a,F,j_{1}}C_{a,F}[j_{1},j_{2}]\sigma_{a,F,j_{2}}$$

**Additive** × **dominance covariances:**


$$\begin{array}{rl} cov(M[F,j_{1}]a_{\;j_{1}},W[F,j_{2}]d_{\;j_{2}}) & =\sigma_{a,F,j_{1}}C_{a,F}[j_{1},j_{2}]\rho_{F,j_{2}}\sigma_{d,F,j_{2}}\\ cov(W[F,j_{1}]d_{\;j_{1}},M[F,j_{2}]a_{\;j_{2}}) & =\sigma_{d,F,j_{1}}\rho_{F,j_{1}}C_{a,F}[j_{1},j_{2}]\sigma_{a,F,j_{2}} \end{array}$$

**Dominance** × **dominance covariances:**

If $j_{1}\neq j_{2}$:


$$cov(W[F,j_{1}]d_{\;j_{1}},W[F,j_{2}]d_{\;j_{2}})=\sigma_{d,F,j_{1}}\rho_{F,j_{1}}C_{a,F}[j_{1},j_{2}]\rho_{F,j_{2}}\sigma_{d,F,j_{2}}$$

If $j_{1}=j_{2}$:


$$cov(W[F,j_{1}]d_{\;j_{1}},W[F,j_{2}]d_{\;j_{2}})=\sigma_{d,F,j_{1}}$$

This distinction ensures that when $j_{1}=j_{2}$, we do not incorrectly scale the variance by $\rho_{F,j}^{2}$, since there is a direct self-connection in the path diagram (no indirect dependency).

In summary, within-family variance using phased genotypes can be computed as:


(13) $$\begin{array}{rl} \text{Var}(g_{F}) & =\sum_{\;j_{1}=1}^{n_{j}}\sum_{\;j_{2}=1}^{n_{j}}[\sigma_{a,F,j_{1}}C_{a,F}[j_{1},j_{2}]\sigma_{a,F,j_{2}}\\ & \;+\sigma_{a,F,j_{1}}C_{a,F}[j_{1},j_{2}]\rho_{F,j_{2}}\sigma_{d,F,j_{2}}\\ & \;+\sigma_{d,F,j_{1}}\rho_{F,j_{1}}C_{a,F}[j_{1},j_{2}]\sigma_{a,F,j_{2}}\\ & \;+\;\sigma_{d,F,j_{1}}\rho_{F,j_{1}}C_{a,F}[j_{1},j_{2}]\rho_{F,j_{2}}\sigma_{d,F,j_{2}}]\\ & \;+\sum_{\;j=1}^{n_{j}}[\sigma_{d,F,j}-\sigma_{d,F,j}\rho_{F,j}C_{a,F}[j,j]\rho_{F,j}\sigma_{d,F,j}] \end{array}$$

All equations above have been developed for single-trait analysis. Extension to multi-trait is available in Appendix B.

### Proportion of additive standard deviation lost

Controlling genetic diversity is central to genomic mating. A simple, interpretable metric helps tune constraints and makes explicit how selection trades short-term response for long-term potential. Although inbreeding rate is commonly used, its accurate estimation typically relies on pedigrees (Wang 2014; Endelman 2025) and does not directly translate into the expected reduction in genetic gain. We therefore define the *proportion of additive standard deviation lost* (*PropSD*), which uses only genomic information and links directly to long-term gain because the breeder’s equation scales linearly with additive standard deviation.


(14) $$PropSD=1-\frac{\sigma_{a_{m}}}{\sigma_{a}},$$

where $\sigma_{a}$ and $\sigma_{a_{m}}$ are the additive standard deviations for the available parental pool and the selected parents of the mating plan, respectively. A naive approach would compute $\sigma_{a}$ and $\sigma_{a_{m}}$ as the sample standard deviations of their genomic estimated additive values. However, this requires marker effects, which are extremely difficult to estimate accurately in quantitative traits (Rice et al. 2008; Korte and Farlow 2013; Ramstein et al. 2019; Clauw et al. 2024). Thus, in the naive approach, it is likely that small effects are wrongly assigned to some important positions, minimizing their impact on the value of *PropSD* and removing any penalization for the fixation of undesired alleles in these loci. To avoid dependence on marker effects, we assume $u∼N(0,G\sigma_{a^{*}}^{2})$, where *G* is a genomic relationship matrix with the associated variance component $\sigma_{a^{*}}^{2}$ and u is a vector of additive values. The expected sample standard deviation of u from a random sample can be written as:


(15) $$\begin{array}{rl} E(s_{a}) & =\beta^{1/2}\frac{\Gamma (\alpha +0.5)}{\Gamma (\alpha )}\\ \alpha & =\frac{E(s_{a}^{2}{)}^{2}}{var(s_{a}^{2})};\beta =\frac{var(s_{a}^{2})}{E(s_{a}^{2})}\\ E(s_{a}^{2}) & =\left[\frac{tr(G)}{n}-\frac{1^{T}G1}{n^{2}}\right]\left(\frac{n}{n-1}\right)\\ var(s_{a}^{2}) & =\frac{1^{T}\Sigma_{2}1}{(n-1{)}^{2}}\\ \Sigma_{2}[i,j] & =2(\Sigma_{1}[i,j]{)}^{2}\\ \Sigma_{1}[i,j] & =G[i,j]+SEM^{2}-\frac{G[i,1\;:\;n]1}{n}-\frac{G[j,1\;:\;n]1}{n}\\ SEM & =\frac{(1^{T}G1{)}^{1/2}}{n} \end{array}$$

Where Γ(⋅) refers to the gamma function, tr(⋅) refers to the trace of a matrix, and *n* is the number of rows or columns in *G*. A detailed mathematical derivation of Equation (15) is available in Appendix C.

$E(s_{a})$ can be used as an estimator for $\sigma_{a}$. We can similarly estimate $\sigma_{a_{m}}$ from $E(s_{a_{m}})$, computed using Equation (15) with $G_{m}$ instead of *G*. This allows us to estimate *PropSD* as:


$$PropSD=1-\frac{E(s_{a_{m}})}{E(s_{a})}$$

While *PropSD* is the primary method for controlling diversity loss in MateR, the inbreeding rate and standard error of the mean are also considered. More details are provided in Appendices D and E.

### Objective function for the optimization

For each family *F*, we define usefulness as


(16) $$U_{F}=E(g_{F})+i_{F}\;r\;\sqrt{\text{Var}(g_{F})},$$

where $E(g_{F})$ and $\text{Var}(g_{F})$ are obtained from Equations (4) and (7); *r* is the prediction accuracy associated with the marker effects employed, and $i_{F}$ is the within-family selection intensity, which can be computed as a function of the proportion of individuals selected within the family. In the optimization process, it is often desired to replicate a cross, i.e. mating two parents more than once to generate more offspring, which increases $i_{F}$. This makes the value of $i_{F}$ dependent on the mating plan, which results in a non-convex optimization problem.

For a mating plan *m* producing $n_{F}$ families, we define fitness as the average usefulness across all families:


(17) $$Fit_{m}=\;\frac{1}{n_{F}}\sum_{F=1}^{n_{F}}U_{F}=\;\frac{1}{n_{F}}\sum_{i=F}^{n_{F}}\left[E(g_{F})+i_{F}r\sqrt{var(g_{F})}\;\right]$$

The optimization problem is then:


(18) $$\begin{array}{c} \underset{m}{\mathrm{argmax}}\;Fit_{m}\\ \text{subject to}:\;PropSD<PropSD_{cutoff} \end{array}$$

where *PropSD* follows Equation (14) and $PropSD_{\mathrm{cutoff}}$ is a user-specified threshold on the acceptable loss of $\sigma_{a}$.

## Materials and methods

### Optimization algorithm

Given the objective in Equation (18), we maximize it with TrainSel (Akdemir et al. 2021), a hybrid heuristic that blends a genetic algorithm with simulated annealing (Akdemir et al. 2021). We ruled out convex optimization and integer programming because the fitness landscape is non-convex and includes discrete replication decisions. The optimization procedure is:

1. Pre-compute the family mean and within-family variance for all possible crosses.
2. Initialize the TrainSel heuristic by creating random mating plans.
3. For each mating plan considered by TrainSel:
  (3.1) Compute *PropSD*. If it exceeds the user threshold, set a very small value for $Fit_{m}$ and jump to step 3.4.
  (3.2) Similarly, enforce any other constraints.
  (3.3) Compute $Fit_{m}$ from Equation (17).
  (3.4) The mating plans with the highest $Fit_{m}$ are classified as elite, and the rest are discarded. Mating plans with lower $Fit_{m}$ may also be accepted during simulated annealing steps.
  (3.5) If convergence or the maximum number of iterations is reached, stop. Otherwise, create new mating plans by applying mutation and crossover operators to the elite solutions and go back to step 3.

MateR supports numerous constraints for step 3.2. To maximize versatility, MateR differentiates between a family and a cross. A family is the set of offspring obtained by crossing two parents. However, these two parents may be crossed more than once. The more crosses that are performed for a given family, the larger the number of offspring for that family. This framework allows for the inclusion of the following constraints:

- Fix the total number of crosses (is often determined by the available budget).
- Enable or disable repeated crosses per family. If it is disabled, there is always exactly one cross per family.
- Fix the number of families to be represented (optional).
- Impose parent-level bounds: minimum and/or maximum number of crosses per parent (optional).
- Specify allowed and forbidden crosses between any pair of parents (optional).
- Include a set of testers (optional); the algorithm then optimizes parental matings to maximize the performance of the hybrids resulting from crossing the progeny with the testers, accounting for general combining ability (GCA) and specific combining ability (SCA).

To our knowledge, MateR is the first genomic mating software to natively optimize mating plans conditional on a user-specified tester set, a feature that is particularly valuable for hybrid breeding.

A detailed guide to MateR usage is available in https://github.com/TheRocinante-lab/MateR.

### Simulated datasets

We simulated one diploid dataset and one autotetraploid dataset. These serve as the MateR example data and were used to validate the proposed equations.

Each dataset contained 1,000 loci distributed across 8 chromosomes of 150 centimorgans (cM) each. For every chromosome, locus allele frequencies were sampled independently. We then imposed a target autocorrelation of 0.95 between adjacent loci along the genetic map. When the maximum feasible correlation between two loci was smaller than 0.95 due to their allele frequencies, the target was reduced to the largest value compatible with those frequencies. Founder haplotypes were sampled iteratively to satisfy the frequency and LD targets, yielding populations with strong linkage disequilibrium, by design, to rigorously probe LD-sensitive variance formulas.

In the diploid population, we created two subpopulations (heterotic-pool analogs), each with 50 fully-homozygous lines. For each subpopulation, we first simulated 200 haplotypes, performed 50 random crosses, and converted the resulting progeny to doubled haploids. Allele frequencies and LD patterns were simulated independently for each subpopulation.

In the autotetraploid dataset, we simulated a single subpopulation with 400 founders and carried out 100 random crosses, producing 100 non-fully homozygous genotypes.

Finally, we simulated marker effects for two traits, Yield (YLD) and maturity (MAT). Marker effects followed the genotypic parameterization and were drawn from a multivariate normal distribution with correlation 0.3 between traits.

### Validation of family mean and variance

To validate the accuracy of the predicted family mean and within-family variance, we sampled a random pair of parents, simulated $10^{4}$ progeny per cross, and took the sample mean and variance of the simulated progeny as the true values for that family. We then compared these values to predictions from MateR, and from other genomic mating software, SimpleMating (Peixoto et al. 2025), predCrossVar (Wolfe et al. 2021), and Genomic Mating (Akdemir and Isidro y Sánchez 2016). Method names and the corresponding linkage disequilibrium (LD) assumptions are summarized in Table 1. As performance metrics, we computed i) Pearson correlation (*accuracy*) and ii) normalized root mean square error (NRMSE = RMSE divided by the standard deviation of the true values). The entire procedure was repeated for 500 independent crosses.

**Table 1.** Name used for each estimator of family mean and variance, classified by how LD is handled and by the software used.

Method	MateR	SimpleMating	predCrossVar	GenomicMating
Family Mean	MateR	SM	pCV	GM
Family Variance, independent loci (1)	MateR_Ind	-	-	GM
Family Variance, approximate LD (2)	MateR_Approx	SM_Approx	-	-
Family Variance, phased genotypes (3)	MateR_Full	SM_Full	pCV	-

The variance estimators correspond to the three formulations derived in the *Mathematical Development* section: (1) Independent loci (_Ind): Assumes zero LD between markers (correlation matrix $C_{F}$ is an identity matrix); variance is computed as the sum of individual locus variances. (2) Approximate LD (_Approx): Estimates within-family LD by propagating the covariance structure of the parental population; uses unphased marker scores. (3) Phased genotypes (_Full): Computes exact LD and covariance using phased parental haplotypes. For GenomicMating, we used only the approaches described in the original publication to avoid redundancy with later updates that are very similar to SimpleMating.

We evaluated five breeding schemes:

- **DH:** line breeding program with double haploids.
- **ILs:** line breeding program with 3 selfing cycles before yield trials.
- **Hybrid_DH:** hybrid breeding program with double haploids.
- **Hybrid_ILs:** hybrid breeding program with 3 selfing cycles before crossing with testers.
- **Clonal:** clonal breeding program, no selfing cycles.

**Fig. 2.**
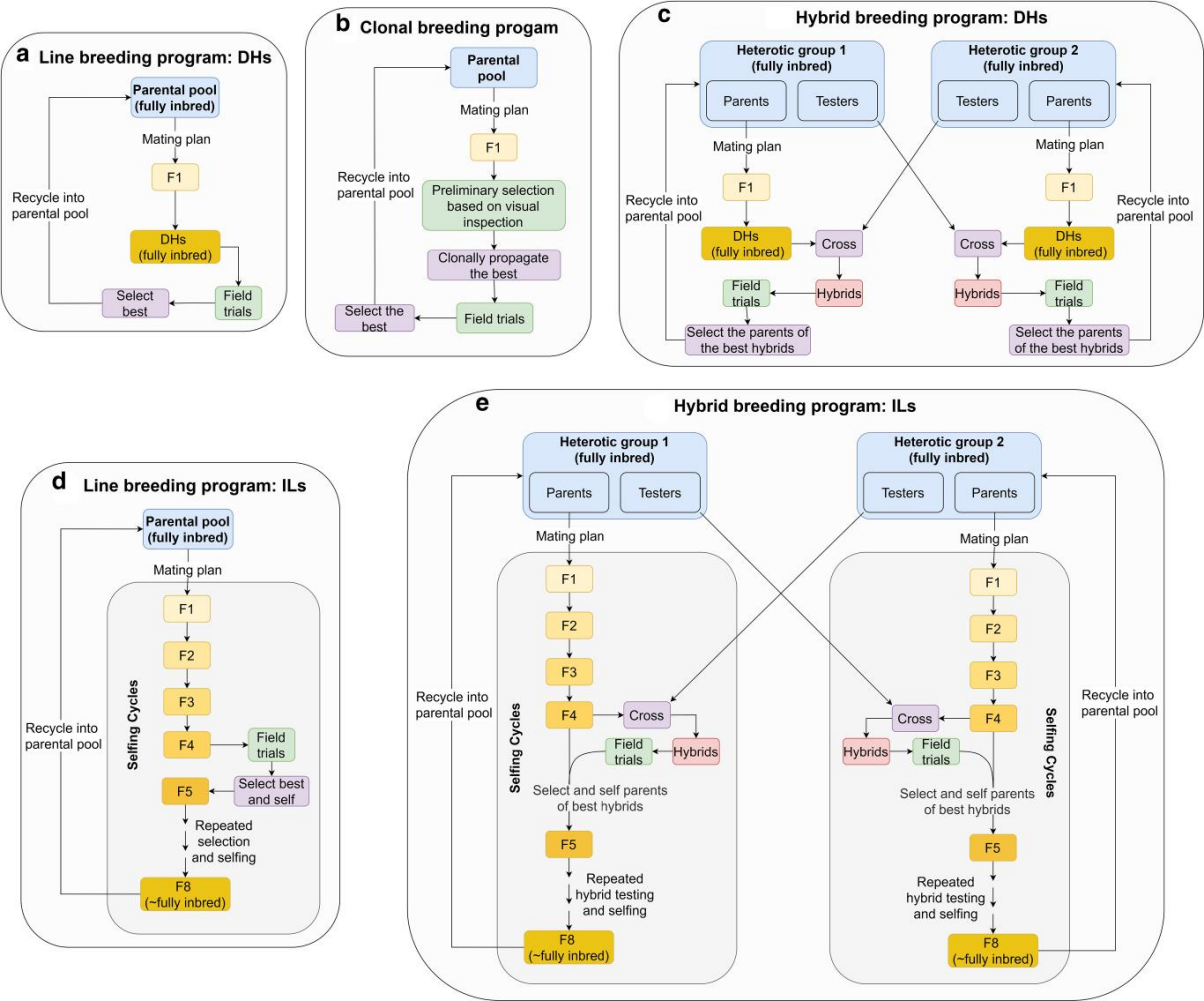
Scheme of a typical breeding program for each of the five breeding approaches tested in this work: a) DH; b) Clonal; c) Hybrid_DH; d) ILs; e) Hybrid_ILs. ILs stands for inbred lines and DHs for double haploids. Please note that these figures only contain the broad structure of each program, and within each type numerous possible variants with differing details are possible.

A detailed overview of each type of breeding program is available in Fig. 2. All schemes were implemented in the diploid population, and non-double-haploid programs were also implemented in the autotetraploid population, yielding a total of 8 scenarios. Not all methods in Table 1 apply to every scenario because some packages support only a subset of breeding schemes.

Within each scenario, we randomly sampled 250 loci as QTL and defined the offspring’s true genotypic values (TGVs) from the corresponding QTL effects. Predictions of family mean and family variance were produced under four genomic prediction settings:

- **QTL_true**: the true QTL effects were used.
- **QTL_1**: estimated QTL effects were obtained. These effects were different from the true QTL effects but resulted in perfect predictions of the genome-wide genotypic values of the parental lines (although predictions were no longer perfect for the offspring).
- **QTL_0.7**: we used estimated QTL effects such that genomic estimated genotypic values (GEGVs) of parents had a correlation of 0.7 with TGVs.
- **Markers_0.7**: marker effects were obtained for the 750 non-QTL loci. These marker effects resulted in GEGVs of the parents that presented a correlation of 0.7 with the TGVs.

### Validation of diversity constraint

We tested whether *PropSD* accurately reflects the expected reduction in additive standard deviation. For both the diploid and autotetraploid datasets, we formed parental samples of sizes 5, 20, 100, and 200 *with replacement*, repeating each size 20 times (80 samples per dataset). Additive genetic values were modeled as u∼N(0,G), with G being the genomic additive relationship matrix of the full parental pool.

Within each parental sample *i*, we drew $10^{5}$ sets of marker effects. Therefore, for each draw *j*, we had a vector of additive values for the parental pool ($u_{\;j}$) and another vector for the sampled parents ($u_{i,j}$). Next, we computed the realized loss of additive variability as


(19) $$PropSD_{true,i,j}=1-\frac{sd(u_{i,j})}{sd(u_{j})}$$

We then compared the predicted $PropSD_{i}$ from Equation (14) with the Monte Carlo average, i.e. $\frac{\sum_{\;j=1}^{10^{5}}PropSD_{true,i,j}}{10^{5}}$.

Because random sampling underestimates the coupling between loci induced by directional selection, we additionally evaluated *PropSD* under selection. Using MateR, we optimized mating plans subject to $PropSD_{\mathrm{cutoff}}\in \{0.001,0.01,0.02,0.03,0.04,0.05\}$, and for each cutoff, we generated 100 independent sets of marker effects used for optimization. We compared predicted and realized *PropSD*, computing realized loss from both (i) the *genetic* standard deviation (captures LD) and (ii) the *genic* standard deviation (excludes LD) to examine Bulmer’s variance depletion under selection (Bulmer 1971).

## Results

Here, we evaluated how predictions for family mean, within-family standard deviation, and *PropSD* agreed with their simulated true values across ploidy levels and scenarios.

### Family mean

**Fig. 3.**
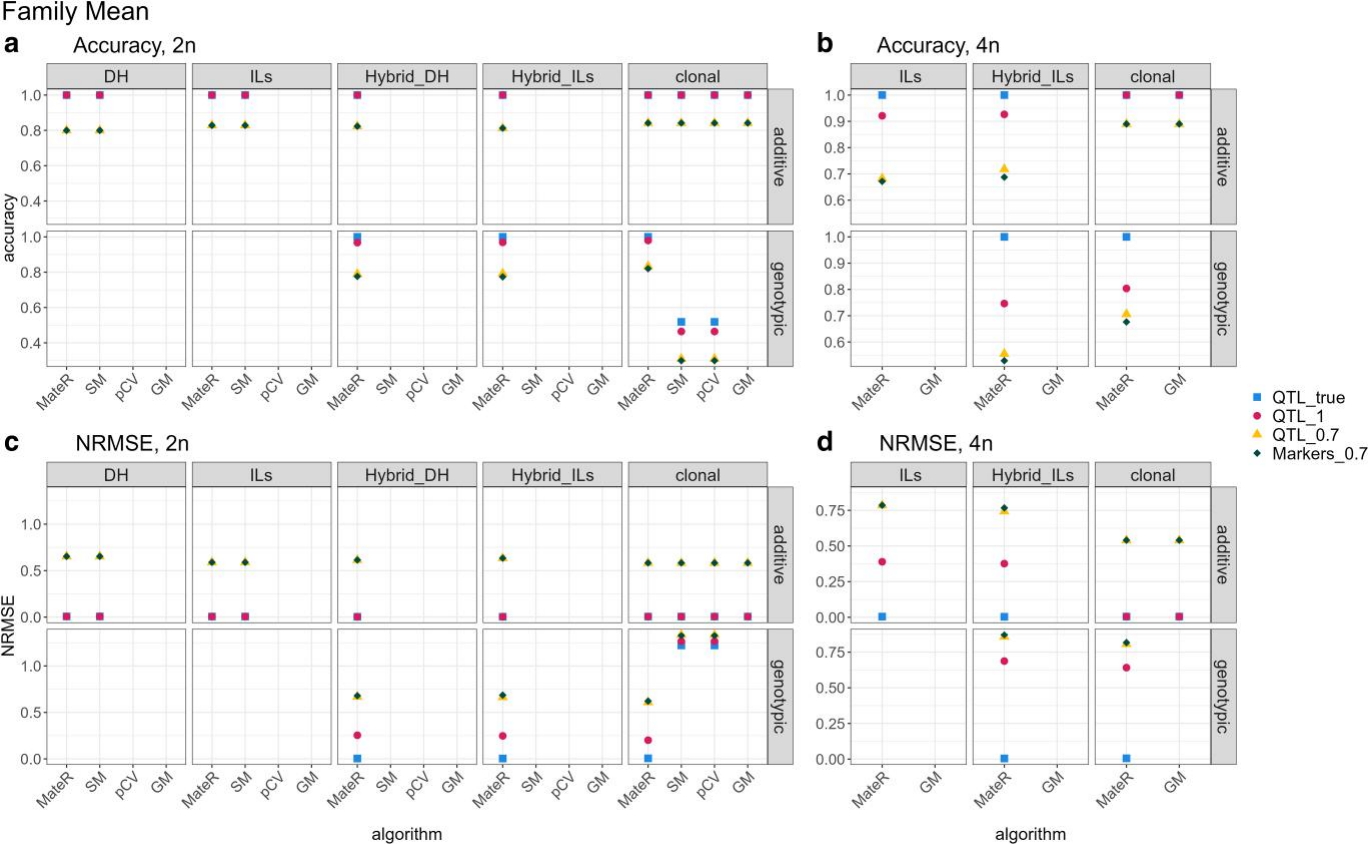
Accuracy (Pearson’s *r*; a,b) and error (NRMSE; c,d) between predicted and true family means across software packages. Panels show diploids (2n; a,c) and autotetraploids (4n; b,d). Within each panel, the top row uses additive values; the bottom row uses genotypic values (additive+dominance). Facet columns are population types (DH, ILs, Hybrid_DH, Hybrid_ILs, clonal). The *x*-axis lists the algorithms used to compute family means (MateR, SM, pCV, GM). Colors/shapes indicate how the effects used for prediction were obtained: **QTL_true**; true QTL effects; **QTL_1**; estimated QTL effects that exactly reproduce parental genome− wide genotypic values (perfect for parents, not necessarily off spring); ▴  **QTL_0.7**; estimated QTL effects whose parental genomic estimated genotypic values (GEGV s) correlate 0.7 with true genotypic values (TGVs); ⧫  **Markers_0.7**; marker effects fitted on the 750 non− QTL loci producing parental GEGVs with correlation 0.7 to TGVs. Blank facets indicate scenarios where the method is not applicable, not implemented in the compared software, or where dominance effects are absent∕irrelevant.

Across additive values, all packages performed identically and achieved perfect accuracy when the true QTL effects were known (Fig. 3a,b). For genotypic values, MateR was superior; it uniquely reached perfect accuracy and was the only software that operated across all scenarios, including the testcross setting in which we predicted the mean of hybrids produced by crossing a family with a fixed tester.

The scenario QTL_1 used QTL effects that were distributed differently from the truth while preserving identical genome-wide predictions in the parental population. Its impact on family-mean accuracy and NRMSE was negligible, with only the non-clonal scenarios for autotetraploids presenting a noticeable drop in accuracy.

Scenarios QTL_0.7 and Markers_0.7 targeted a predictive accuracy in the parental pool of ≈0.7. In diploids, family-mean accuracy consistently exceeded this baseline (around 0.8). In autotetraploids, accuracy was less uniform and ranged from 0.55 to 0.90, with higher values in the clonal program and for additive effects.

As expected, NRMSE mirrored these trends (Fig. 3c,d). Most importantly, when predicted and true values were perfectly correlated, NRMSE equaled zero, indicating unbiased predictors.

### Within-family standard deviation

**Fig. 4.**
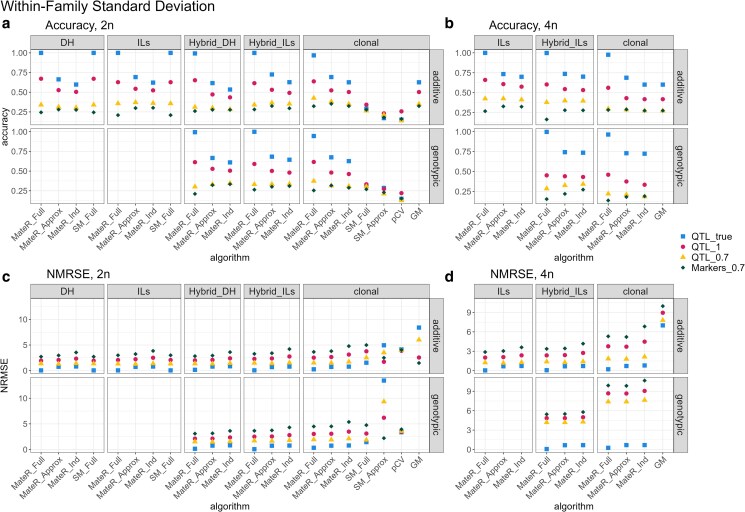
Within-family standard deviation. Agreement between predicted and true within-family SD across software packages. a,b) Pearson correlation; c,d) NRMSE. Panels show diploids (2n; a,c) and autotetraploids (4n; b,d). Within each panel, the top row uses additive values; the bottom row uses genotypic values (additive+dominance). Facet columns are population types (DH, ILs, Hybrid_DH, Hybrid_ILs, clonal). The *x*-axis lists the algorithm (MateR_Full, MateR_Approx, MateR_Ind, SM_Full, SM_Approx, pCV, GM). Colors/shapes encode how effects for prediction were obtained: QTL_true; true QTL effects; **QTL_1**; estimated QTL effects that exactly reproduce parental genome-wide genotypic values; ▴  **QTL_0.7**; estimated QTL effects whose parental GEGVs correlate 0.7 with true genotypic values (TGVs); ⧫  **Markers_0.7**; marker effects fitted on the 750 non-QTL loci giving parental GEGV–TGV correlation 0.7. Blank facets indicate scenarios where the method is not applicable, not implemented in the compared software, or where dominance effects are absent/irrelevant.

MateR_Full (phased genotypes as input) was the only method that achieved perfect or near-perfect prediction of within-family standard deviation across all scenarios (Fig. 4). SM_Full matched this performance only for additive values with diploid and fully homozygous parental lines–specifically, the DH and ILs scenarios in Fig. 4a. In the diploid clonal scenario, MateR_Full, SM_Full, and pCV all used phased genotypes, yet only MateR_Full translated these inputs into perfect predictions for both additive and genotypic standard deviation.

When phased genotypes were not available (MateR_Approx), accuracy dropped to around ∼0.65–0.75 with known true QTL effects (Fig. 4a,b). Assuming marker independence (MateR_Ind) produced only a modest additional decrease and showed similar trends for NRMSE (Fig. 4c,d).

Degrading the quality of QTL or marker effects (QTL_1, QTL_0.7, Markers_0.7) reduced accuracy, increased NRMSE, and narrowed the advantage of MateR_Full over MateR_Approx and MateR_Ind. Under the most realistic setting, Markers_0.7, accuracy hovered around 0.25 and NRMSE frequently approached or exceeded 3, indicating systematic underestimation by more than three standard deviations. In this setting, MateR_Approx often showed slightly higher accuracy and comparable NRMSE to MateR_Full. MateR_Ind exhibited similar or marginally higher accuracy than MateR_Approx , but with a larger NRMSE. Among non-MateR methods, SM_Full performed best and was comparable to MateR_Full in its three applicable scenarios (diploid ILs, DH, and clonal). In contrast, SM_Approx and pCV, usable only in the diploid clonal scenario, showed very low accuracy even with true QTL. SM_Approx also had an extremely large NRMSE, while pCV produced reasonable NRMSE but frequently predicted negative variances, which is problematic. Finally, GM applied only to additive values in clonal programs (both ploidies). In theory, GM and MateR_Ind should be exactly equivalent, but we found that the current version of GM (2.0) presents a coding bug that inflates the SD by a factor of two. As a result, it presents the same accuracy as MateR_Ind but with a much larger NRMSE (Fig. 4).

### Proportion of standard deviation lost

**Fig. 5.**
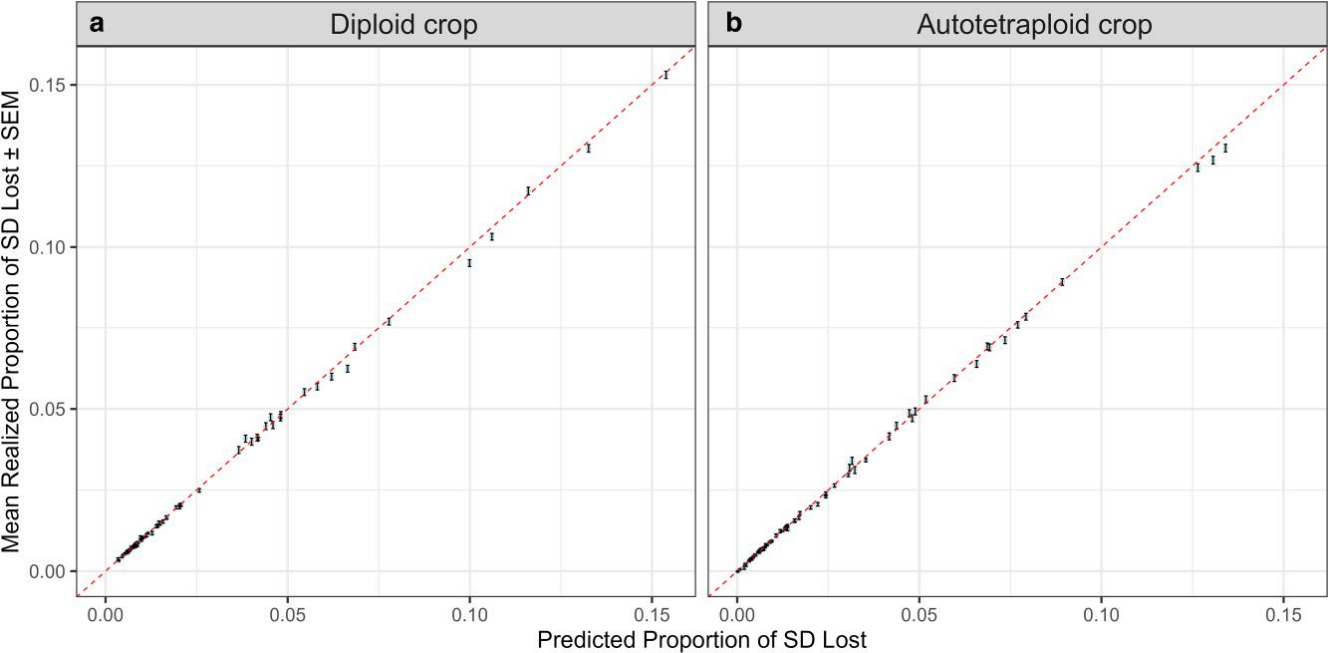
Correspondence between the *predicted* (horizontal axis) and the *true* (vertical axis) *PropSD* for diploid a) and autotetraploid b) datasets under **random sampling**. Each point belongs to a different random sample of selected parental lines and and shows the average across $10^{5}$ sets of random marker effects. Error bars indicate the standard error of the mean (SEM). The dashed line marks perfect correspondence (identity).

Under random sampling, *PropSD* computed with Equation (15) was extremely accurate and presented an almost perfect correlation with the average true proportion of the additive standard deviation lost, as shown in Fig. 5. This was true for both diploid and autotetraploid crops.

**Fig. 6.**
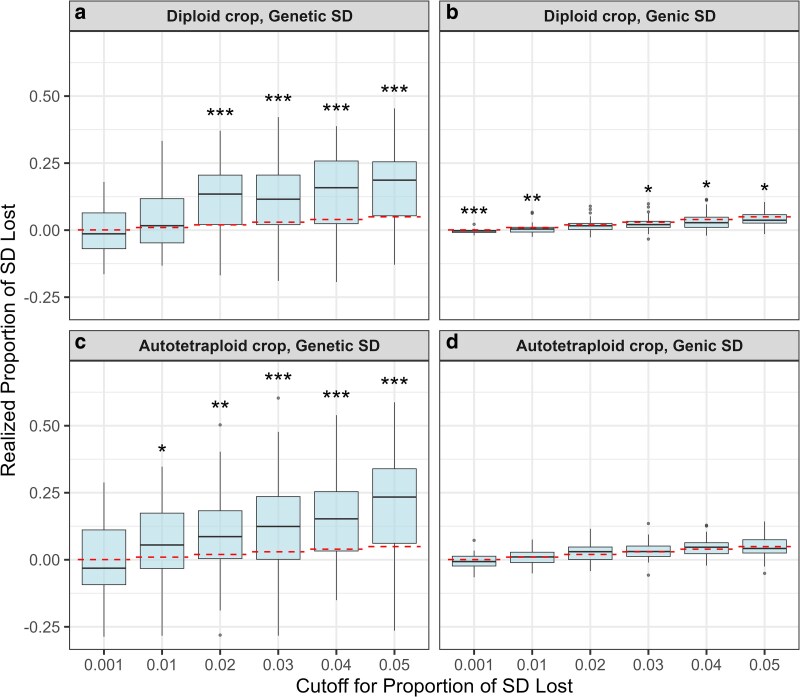
Correspondence between predicted and true *PropSD* under *selection* for diploid a,b) and autotetraploid c,d) datasets. Horizontal dashed lines indicate the average *predicted PropSD* for the selected parents. Boxplots show the *realized* proportion of a,c) *genetic* standard deviation lost (includes LD) and b,d) *genic* standard deviation lost (LD-free). Asterisks denote the significance of paired Wilcoxon tests comparing predicted and realized values (* for 0.01<p<0.05, ** for 0.001<p<0.01, *** for p<0.001). Parametric tests were avoided because their assumptions were not met

Under selective pressure, the true proportion of genetic standard deviation lost was consistently and significantly larger than the predicted value (Fig. 6a,c). By contrast, the predicted *PropSD* aligned much more closely with the realized loss of *genic* standard deviation (Fig. 6b,d). In diploids, the realized genic loss tended to be lower than the predicted *PropSD*, whereas, in autotetraploids, the two were similar on average. Furthermore, genic standard deviation (Fig. 6b,d) presented much lower dispersion than genetic standard deviation, as evidenced by the narrower boxplots (Fig. 6b,d vs. a,c).

## Discussion

This section focuses on interpreting the results from the previous section. However, it is important to note that a detailed showcase of the application of MateR to multi-generation breeding programs is available in Metwally et al. (2025).

### Family mean

Under pure additivity, predicting the family mean is trivial, as it corresponds to the mid-parental value. Kinghorn (2011), Akdemir and Isidro y Sánchez (2016), Wolfe et al. (2021) and Peixoto et al. (2025) use this approach to achieve perfect accuracy when the true QTL effects are known, as seen in Fig. 3. Our approach (Equation (4)) is equivalent and yields identical results for any set of QTL or marker effects.

When dominance is relevant, existing software relies on Equation (2) (Wolfe et al. 2021; Peixoto et al. 2025). This formulation is restricted to F1 generations and requires genotypic parameterization, which limits its applicability. Furthermore, Fig. 3 shows that it is not fully accurate even when the true QTL effects are known, which was unexpected. Further investigation revealed that Equation (2) outputs wrong results, as illustrated in Supplementary File 1.

Finally, family mean predictions proved robust to QTL misidentification. In Fig. 3, the QTL_true scenario was extremely similar to QTL_1, and QTL_0.7 was almost identical to Markers_0.7. These results indicate that overall genome-wide prediction accuracy is the primary determinant of family mean accuracy, whereas precise knowledge of the loci distribution plays a secondary role.

### Within-family standard deviation

Predicting within-family standard deviation is harder than predicting the family mean because correlations among loci contribute to the variance. Exact prediction therefore requires phased parental haplotypes to compute LD. In our benchmarks, MateR_Full, which uses phased haplotypes, was the only approach that consistently achieved near-perfect accuracy; SM_Full performed similarly only when dominance was absent and the parents were fully homozygous (Fig. 4). SM_Full (Peixoto et al. 2025), based on the equations of Lehermeier et al. (2017b), was robust in the scenarios in which they apply. Our equations achieve comparable performance while also generalizing to dominance and autopolyploids.

Accuracy depended strongly on the fidelity of the effect map. Perfect accuracy was attainable only when the true QTL were known; it dropped markedly even in the QTL_1 scenario, where causal effects were redistributed, but genome-wide prediction accuracy remained ideal. This sensitivity follows directly from Equations (6) and (7): within-family variance aggregates cross-locus covariances, with weights proportional to locus effects, so the genomic placement of effects is decisive. In the most realistic setting (Markers_0.7), SD prediction accuracy was typically ≈0.25, which helps explain why the usefulness criterion underperforms mean-based selection in some studies (Wang et al. 2025). Nevertheless, modeling SD with accuracy ≈0.25 should be preferable to ignoring it, consistent with positive results reported elsewhere (Lehermeier et al. 2017b). Additional insights on the factors that determine whether the usefulness criterion is beneficial in a given population can be obtained with the equation for *Usefulness Importance* developed in Metwally et al. (2025).

Please note that predicting within-family variance has additional value apart from the implementation of the usefulness criterion. For instance, it can be used to determine the desired progeny size. When the average within-family variance is large compared to the variability between family means, it is recommended to generate few families with a large progeny, as a lot of gain can be obtained by applying a high selection intensity within each family. The opposite is true when within-family variance is low. Even the rough estimates provided by MateR under realistic circumstances (Fig. 4) can be very valuable for this purpose. In the Markers_0.7 scenario, MateR_Approx actually slightly outperformed MateR_Full, while being simpler, faster, and not requiring phased genotypes. We therefore recommend MateR_Approx as the default, reserving MateR_Full for cases with high-quality phased haplotypes and accurately estimated marker effects.

Overall, our results indicate that the current bottleneck for variance prediction is not the formulae but the availability of realistic marker effect landscapes for quantitative traits (Rice et al. 2008; Korte and Farlow 2013; Ramstein et al. 2019; Clauw et al. 2024), as neither MateR nor other alternatives are robust to marker effect misspecification and suffer large accuracy drops as a result.

### Proportion of standard deviation lost

Balancing short-term gains with the long-term diversity benefits from an interpretable metric. Since $\sigma_{a}$ scales the expected response to selection, a given per-cycle value of *PropSD* implies that, assuming fixed selection intensity and accuracy, the next-cycle response satisfies $R_{t+1}=(1-PropSD)R_{t}$. This framing enables the explicit, quantitative evaluation of long-term consequences for any chosen cutoff on *PropSD*. For such use to be reliable, predicted *PropSD* must accurately track the realized loss of genetic variability. Critically, our formulation of *PropSD* does not rely on estimated marker effects, ensuring its robustness against marker effect misspecification.

Under random sampling, the predicted *PropSD* matched the observed average across replicates (Fig. 5), validating Equation (14). Under selection, however, the reduction in genetic SD exceeded predictions (Fig. 6). This pattern is consistent with the Bulmer effect (Bulmer 1971): selection rapidly induces negative LD among QTL, depleting genetic variance beyond genic expectations. Our simulated base population lacked prior selection, so LD built up sharply in the first cycle. In contrast, long-running breeding programs are typically nearer Bulmer equilibrium, so deviations from Equation (14) should be smaller or non-existent. Supporting this interpretation, the observed loss of *genic* SD (unaffected by LD) was similar to or slightly lower than the predicted *PropSD*. In summary, *PropSD* is unable to track the Bulmer effect, which can cause it to deviate from the realized loss in genetic SD, especially in early cycles when the population is far from Bulmer equilibrium or after sudden changes in selection intensity. It is very robust otherwise.

Multi-cycle evaluations in Metwally et al. (2025) show that *PropSD* performed well for controlling variability in long-term breeding programs. As a rule of thumb, *PropSD* near 0.04-0.05 is recommended, but the optimal *PropSD* value may vary for different breeding programs, requiring specific tuning.

### General considerations

A detailed comparison of existing genomic mating software with MateR is provided in Supplementary File 2. A key strength of MateR is its versatility. It accommodates diverse breeding schemes, including hybrid pipelines. In reciprocal recurrent selection, it identifies the optimal mating plan within a heterotic pool to maximize hybrid performance when progeny are testcrossed to the opposite pool, a functionality that, to our knowledge, is unique.

MateR also optimizes the number of crosses allocated to each parental pair. Replicating a cross increases family size, thereby increasing within-family selection intensity and usefulness. Crucially, the benefit scales with within-family variance; families with near-zero variance gain little from additional sampling. Thus, cross-replication decisions should be guided by reliable variance prediction.

MateR can be readily extended to animal breeding by setting ploidy to diploid and disabling both self-fertilization and cross replication. The framework supports essential mating constraints, including limiting females to a single mating, imposing upper or lower mating bounds on males, and prohibiting specific male–female pairings. Expected progeny size can also be specified, accommodating differences among animal species.

## Supplementary Material

iyag013_Supplementary_Data

## Data Availability

The MateR package and the example datasets are available at https://github.com/TheRocinante-lab/MateR The code used to generate the results is available at https://github.com/TheRocinante-lab/Publications/tree/main/2026/Fernandez_Gonzalez_et_al_MateR. Any additional resources are provided in Files S1–S2. Supplemental material available at GENETICS online.

## Appendix A: Genotypic frequencies and extension to polyploids

### Diploid crop, prediction of genotypic frequencies

In a diploid crop, for a single gene with two alleles, *A* and *a*, there are three possible genotypes: *AA*, *Aa*, and *aa*. When we cross two parents, we want to predict the frequencies of each genotype ($P_{AA}$, $P_{Aa}$, $P_{aa}$) in their offspring family. These frequencies can be obtained from basic Mendelian genetics. We also need to extend them for self-pollination (selfing) over multiple generations. Selfing changes the genotypic frequencies in a predictable way:

- The heterozygous genotype *Aa* becomes less frequent by half each generation.
- Each homozygous genotype (*AA* and *aa*) increases by one quarter of the *Aa* frequency from the previous generation.

Mathematically:

$$\begin{array}{rl} P_{AA}^{\left(n\right)} & =P_{AA}^{\left(n-1\right)}+0.25\cdot P_{Aa}^{\left(n-1\right)}\\ P_{Aa}^{\left(n\right)} & =0.5\cdot P_{Aa}^{\left(n-1\right)}\\ P_{aa}^{\left(n\right)} & =P_{aa}^{\left(n-1\right)}+0.25\cdot P_{Aa}^{\left(n-1\right)} \end{array}$$

After rearranging and simplifying, we can generalize the genotype frequencies after *n* generations of selfing for different parental combinations, as shown in Table A1. Double haploids correspond to the limit when *n* tends to infinity.

**Table A1.** Genotypic frequencies in a diploid locus with alleles *A* and *a*, for different parental combinations after *n* cycles of selfing.

P1	P2	AA (n=0)	Aa (n=0)	aa (n=0)	AA (any *n*)	Aa (any *n*)	aa (any *n*)
AA	AA	1	0	0	1	0	0
aa	aa	0	0	1	0	0	1
AA	aa	0	1	0	$\frac{1}{2}-(\frac{1}{2}{)}^{n+1}$	$(\frac{1}{2}{)}^{n}$	$\frac{1}{2}-(\frac{1}{2}{)}^{n+1}$
Aa	Aa	$\frac{1}{4}$	$\frac{1}{2}$	$\frac{1}{4}$	$\frac{1}{2}-(\frac{1}{2}{)}^{n+2}$	$(\frac{1}{2}{)}^{n+1}$	$\frac{1}{2}-(\frac{1}{2}{)}^{n+2}$
AA	Aa	$\frac{1}{2}$	$\frac{1}{2}$	0	$\frac{3}{4}-(\frac{1}{2}{)}^{n+2}$	$(\frac{1}{2}{)}^{n+1}$	$\frac{1}{4}-(\frac{1}{2}{)}^{n+2}$
Aa	aa	0	$\frac{1}{2}$	$\frac{1}{2}$	$\frac{1}{4}-(\frac{1}{2}{)}^{n+2}$	$(\frac{1}{2}{)}^{n+1}$	$\frac{3}{4}-(\frac{1}{2}{)}^{n+2}$

The first two columns contain the genotypes of the two parents. The other columns contain the frequencies of each possible genotype in the offspring.

It is important to note that allopolyploids are normally modeled as diploids. Therefore, Table A1 can also be applied to them.

### Extension to autotetraploids

The equations used for diploid crops can be extended to autopolyploid species, such as autotetraploids, by adapting the genotype states and their expected values. In the case of autotetraploids, we assume **bivalent and non-preferential pairing** during meiosis.

At a biallelic locus *j* in an autotetraploid family *F*, there are five possible genotypes: *AAAA*, *AAAa*, *AAaa*, *Aaaa*, and *aaaa*. The corresponding genotypic frequencies are denoted as:

- $PAAAA_{F,j}$ for genotype *AAAA*
- $PAAAa_{F,j}$ for genotype *AAAa*
- $PAAaa_{F,j}$ for genotype *AAaa*
- $PAaaa_{F,j}$ for genotype *Aaaa*
- $Paaaa_{F,j}$ for genotype *aaaa*

To translate these genotypes into numeric form, we use two types of incidence matrices:

- **Additive incidence matrix:** represents the number of copies of the *a* allele. AAAA=0, AAAa=1, AAaa=2, Aaaa=3, and aaaa=4
- **Dominance incidence matrix:** represents the presence of heterozygosity (non-homozygosity). AAAa=3, AAaa=4, Aaaa=3, while AAAA=0 and aaaa=0. This can be generalized to any ploidy as x(ϕ−x), where *ϕ* is the degree of ploidy and *x* refers to the additive score, i.e. the number of times the *a* allele is present Labroo et al. (2023). This equation only captures the digenic dominance component and discards trigenic or higher-order interactions, which is a common simplification Endelman et al. (2018).

These incidence scores belong to the genotypic parameterization. In the breeding parameterization, for a minor allele frequency in the reference population *p*, we would need to subtract ϕp from the additive scores and use −2(ϕ2)p2+2p(ϕ−1)x−x(x−1) for the dominance scores (Endelman 2023).

Continuing with the genotypic parameterization for the sake of simplicity, and using the same nomenclature as in Equations (3) and (5), we define the expected genotypic value ($\mu_{F,j}$) and variance ($\sigma_{F,j}^{2}$) at locus *j* for family *F* as follows:

(A1 )
$$\begin{array}{rl} \mu_{F,j} & =PAAAA_{F,j}(0a_{j}+0d_{j})\\ & \;+PAAAa_{F,j}(1a_{j}+3d_{j})\\ & \;+PAAaa_{F,j}(2a_{j}+4d_{j})\\ & \;+PAaaa_{F,j}(3a_{j}+3d_{j})\\ & \;+Paaaa_{F,j}(4a_{j}+0d_{j}) \end{array}$$

(A2 )
$$\begin{array}{rl} \sigma_{F,j}^{2} & =PAAAA_{F,j}(0a_{j}+0d_{j}-\mu_{F,j}{)}^{2}\\ & \;+PAAAa_{F,j}(1a_{j}+3d_{j}-\mu_{F,j}{)}^{2}\\ & \;+PAAaa_{F,j}(2a_{j}+4d_{j}-\mu_{F,j}{)}^{2}\\ & \;+PAaaa_{F,j}(3a_{j}+3d_{j}-\mu_{F,j}{)}^{2}\\ & \;+Paaaa_{F,j}(4a_{j}+0d_{j}-\mu_{F,j}{)}^{2} \end{array}$$

Equations (4), (7), and (13) remain valid for autotetraploids. The only requirement is to compute the locus-specific means and variances using Equations (A1) and (A2), which account for the additional genotypic classes and dosage effects in tetraploid contexts. These per-locus values are then summed or combined across the genome as in the diploid case.

### Autotetraploid crop, prediction of genotypic frequencies

The genotypic frequencies of the F1 generation in autotetraploid crops are summarized in Table A2. These initial frequencies form the basis for computing the genotypic frequencies in subsequent generations after *n* cycles of selfing. The frequencies from Table A2 are derived from the convolution of two hypergeometric distributions, which represent the probabilities of sampling alleles from the gametes of each parent under random bivalent pairing Serang et al. (2012) and Gerard et al. (2018). While autotetraploids are the only autopolyploids currently supported by MateR, this methodology could be used to extend it to any degree of ploidy.

**Table A2.** Genotypic frequencies in an autotetraploid locus with two alleles (*A* and *a*), obtained when crossing two parents (P1 and P2).

P1	P2	AAAA	AAAa	AAaa	Aaaa	aaaa
AAAA	AAAA	1	0	0	0	0
aaaa	aaaa	0	0	0	0	1
AAAA	AAAa	$\frac{1}{2}$	$\frac{1}{2}$	0	0	0
Aaaa	aaaa	0	0	0	$\frac{1}{2}$	$\frac{1}{2}$
AAAA	AAaa	$\frac{6}{36}$	$\frac{24}{36}$	$\frac{6}{36}$	0	0
AAaa	aaaa	0	0	$\frac{6}{36}$	$\frac{24}{36}$	$\frac{6}{36}$
AAAA	Aaaa	0	$\frac{1}{2}$	$\frac{1}{2}$	0	0
AAAa	aaaa	0	0	$\frac{1}{2}$	$\frac{1}{2}$	0
AAAa	AAAa	$\frac{1}{4}$	$\frac{1}{2}$	$\frac{1}{4}$	0	0
Aaaa	Aaaa	0	0	$\frac{1}{4}$	$\frac{1}{2}$	$\frac{1}{4}$
AAAa	AAaa	$\frac{3}{36}$	$\frac{15}{36}$	$\frac{15}{36}$	$\frac{3}{36}$	0
AAaa	Aaaa	0	$\frac{3}{36}$	$\frac{15}{36}$	$\frac{15}{36}$	$\frac{3}{36}$
AAAa	Aaaa	0	$\frac{1}{4}$	$\frac{1}{2}$	$\frac{1}{4}$	0
AAaa	AAaa	$\frac{1}{36}$	$\frac{8}{36}$	$\frac{18}{36}$	$\frac{8}{36}$	$\frac{1}{36}$
AAAA	aaaa	0	0	1	0	0

The first two columns show the parental genotypes. The next five columns display the frequencies of genotypes in the F1 generation

To compute genotypic frequencies after selfing, we use a selfing transition matrix (*X*), which has the values from Table A3 in autotetraploids, following the concept of the generation matrix from Fisher (1949), Kempthorne (1957) and Workman and Allard (1962). The frequencies in *X* are particular cases of Table A2. *X* is a column-stochastic square matrix where each parental genotype class is acting like a ”state” in a Markov-chain. Its columns contain the conditional probabilities of transitioning from parental to progeny states, with ”time” being the number of selfing cycles. For any parental genotype *j* and any offspring g*enotype i*:

$$\sum_{i=1}^{\phi +1}X_{ij}=\sum_{i}P(\mathrm{progeny}=i|\mathrm{parent}=j)=1$$

Where *ϕ* is the degree of ploidy and the number of possible genotypic classes is therefore ϕ+1. Based on the law of total probability, the overall probability that an offspring ends up being of genotype *i* has to be:

$$P(\mathrm{progeny}=i)=\sum_{\;j}^{\phi +1}P(\mathrm{progeny}=i|\mathrm{parent}=j)\;P(\mathrm{parent}=j)\;$$

For any family *F*, this can be generalized in matrix notation as follows:

$$f_{n,F}=X^{n}f_{0,F}$$

where $f_{0,F}$ is a vector of F1 progeny genotypic frequencies and $f_{n,F}$ is a vector of progeny genotypic frequencies after *n* selfing cycles.

**Table A3.** *X* matrix in table format.

	AAAA	AAAa	AAaa	Aaaa	aaaa
AAAA	1	$\frac{1}{4}$	$\frac{1}{36}$	0	0
AAAa	0	$\frac{1}{2}$	$\frac{8}{36}$	0	0
AAaa	0	$\frac{1}{4}$	$\frac{18}{36}$	$\frac{1}{4}$	0
Aaaa	0	0	$\frac{8}{36}$	$\frac{1}{2}$	0
aaaa	0	0	$\frac{1}{36}$	$\frac{1}{4}$	1

It contains the parental genotypes in the columns, and each row contains the probability of an offspring genotype after selfing the parent.

## Appendix B: Extension of family mean and variances to multi-trait

The equations described before for computing genotypic means and variances at the single-locus level can be readily extended to a multi-trait context. In multi-trait selection, the goal is often to combine information from multiple traits into a single value that reflects overall genotypic value or fitness. This is commonly achieved using a **selection index**.

Let:

- *M* be the n×p matrix of additive marker scores, where *n* is the number of individuals and *p* is the number of markers.
- *W* be the corresponding n×p matrix of dominance scores.
- $a_{t}$ and $d_{t}$ be p×1 vectors of additive and dominance effects, for trait *t*.
- $c_{t}$ be a scalar weight (coefficient derived from economic value or desired gains) assigned to trait *t*, used to combine traits into a single selection index.
- $n_{t}$ be the number of traits included in the index.

The genotypic values for individuals with respect to trait *t* are given by:

$$g_{t}=Ma_{t}+Wd_{t}$$

The multi-trait selection index scores (IS) for all individuals is computed as a weighted linear combination of their genotypic values across traits:

(B1 )
$$IS=\sum_{t=1}^{n_{t}}c_{t}g_{t}=\sum_{t=1}^{n_{t}}c_{t}\left(Ma_{t}+Wd_{t}\right)$$

Using the distributive property of matrix multiplication, this expression simplifies to:

(B2 )
$$IS=M\left(\sum_{t=1}^{n_{t}}c_{t}a_{t}\right)+W\left(\sum_{t=1}^{n_{t}}c_{t}d_{t}\right)$$

We can then define the **multi-trait additive and dominance marker effects** as:

$$a_{IS}=\sum_{t=1}^{n_{t}}c_{t}a_{t},\;d_{IS}=\sum_{t=1}^{n_{t}}c_{t}d_{t}$$

Thus, the multi-trait selection index is:

$$IS=Ma_{IS}+Wd_{IS}$$

This representation reduces the multi-trait selection problem to a **single-trait framework**, where the trait is the index itself. Consequently, we can use the previously defined equations for genotypic mean (Equation (3)) and variance (Equation (5)) by replacing $a_{j}$ and $d_{j}$ with $a_{IS,j}$ and $d_{IS,j}$.

## Appendix C: Mathematical derivation for the proportion of additive standard deviation lost

Let $u∼N(0,G\sigma_{a^{*}}^{2})$ be the additive values for the entire parental pool and $u_{m}∼N(0,G_{m}\sigma_{a^{*}}^{2})$ be the additive values for the selected parents. It is important to note that $\sigma_{a^{*}}^{2}$ is a scaling factor (often estimated with REML) but it has no biological meaning due to the improper scaling of the *G* matrix Fernández-González and Isidro y Sánchez (2025). We will use an asterisk to identify the non-interpretable scaling factors, while variance components with actual biological meaning will be written without the asterisk.

Due to selection, the genetic standard deviation in $u_{m}$ is often lower than that of ***u***. The proportion of standard deviation retained after selection is $\frac{\sigma_{a_{m}}}{\sigma_{a}}$. Thus, the proportion of standard deviation lost (*PropSD*) is $PropSD=1-\frac{\sigma_{a_{m}}}{\sigma_{a}}$. The actual value of the standard deviations in the parental pool ($\sigma_{a}$) and in the selected parents ($\sigma_{a_{m}}$) is unknown, but it is possible to estimate them as the expected standard deviation of an infinite amount of samples from the corresponding multivariate distributions: ${\hat{\sigma }}_{a}=E(s_{a});{\hat{\sigma }}_{a_{m}}=E(s_{a_{m}})$. To obtain these estimators, we will first obtain the expected sample variance:

(C1 )
$$E(s_{a}^{2})=\left[\frac{tr(G\sigma_{a^{*}}^{2})}{n}-\frac{1^{T}G\sigma_{a^{*}}^{2}1}{n^{2}}\right]\left(\frac{n}{n-1}\right)$$

(C2 )
$$var(s_{a}^{2})=SEV^{2}$$

(C3 )
$$E(s_{a_{m}}^{2})=\left[\frac{tr(G_{m}\sigma_{a^{*}}^{2})}{n_{m}}-\frac{1^{T}G_{m}\sigma_{a^{*}}^{2}1}{n_{m}^{2}}\right]\left(\frac{n_{m}}{n_{m}-1}\right)$$

(C4 )
$$var(s_{a_{m}}^{2})=SEV_{m}^{2}$$

Where *n* is the total number of parents, $n_{m}$ is the number of parents selected for the mating plan, *SEV* is the standard error of the variance computed as in Fernández-González and Isidro y Sánchez (2025) from the distribution $u∼N(0,G\sigma_{a^{*}}^{2})$ and $SEV_{m}$ is the standard error of the variance corresponding to $u_{m}∼N(0,G_{m}\sigma_{a^{*}}^{2})$. It is important to note that equations (C1) and (C3) are computed following Searle et al. (1992) and Endelman (2023) with the addition of the correction factor $\frac{n}{n-1}$ to account for the fact that we are computing the expectation of sample variance, not population variance.

Additionally, while it can be tempting to estimate $1-\frac{\sigma_{a_{m}}}{\sigma_{a}}$ as $1-\frac{\sqrt{E(s_{a_{m}}^{2})}}{\sqrt{E(s_{a}^{2})}}$, this is not possible due to the fact that $\sqrt{E(s_{a}^{2})}\neq E(\sqrt{s_{a}^{2}})$. Thus, we need to find a way for computing $E(\sqrt{s_{a}^{2}})=E(s_{a})$. Thankfully, it is known that sample variance follows a gamma distribution. Therefore:

(C5 )
$$s_{a}^{2}∼\Gamma \left(\mu =E(s_{a}^{2}),\sigma^{2}=var(s_{a}^{2})\right)=\Gamma \left(\alpha =\frac{E(s_{a}^{2}{)}^{2}}{var(s_{a}^{2})},\beta =\;\frac{var(s_{a}^{2})}{E(s_{a}^{2})}\right)$$

Where *α* and *β* are the shape and scale parameters of a gamma distribution. As a result, we can compute $E(s_{a})$ from the moment of order 1/2 of a gamma distribution with the following probability density function:

$$f(s_{a}^{2})=\frac{\beta^{\alpha }}{\Gamma (\alpha )}{s_{a}^{2}}^{\left(\alpha -1\right)}e^{\left(-\beta s_{a}^{2}\right)}$$

Where Γ(⋅) refers to the gamma function. $f(s_{a}^{2})$ is defined for all $s_{a}^{2}>0$. Gamma distributions have the property that Γ(α,β)=β⋅Γ(α,1). Thus, if *β* is taken out of the gamma distribution, we can simplify the probability density function to:

$$f(s_{a}^{2})=\beta \cdot \left(\frac{1}{\Gamma (\alpha )}{s_{a}^{2}}^{\left(\alpha -1\right)}e^{\left(-s_{a}^{2}\right)}\right)$$

Therefore, we can define $x=\frac{s_{a}^{2}}{\beta };f(x)=\frac{1}{\Gamma (\alpha )}x^{\left(\alpha -1\right)}e^{\left(-x\right)}$. Next, applying the equation for the moment of order 1/2 of a continuous distribution:

(C6 )
$$\begin{array}{rl} E\left((s_{a}^{2}{)}^{1/2}\right) & =\;E\left((\beta x{)}^{1/2}\right)=\beta^{1/2}E\left(x^{1/2}\right)=\beta^{1/2}\int_{0}^{\infty }x^{1/2}\cdot f(x)\;dx;\\ E(s_{a}) & =\;\beta^{1/2}\int_{0}^{\infty }x^{1/2}\left(\frac{1}{\Gamma (\alpha )}x^{\left(\alpha -1\right)}e^{\left(-x\right)}\right)\;dx;\\ E(s_{a}) & =\;\beta^{1/2}\frac{1}{\Gamma (\alpha )}\int_{0}^{\infty }x^{\left(\alpha -1/2\right)}e^{\left(-x\right)}\;dx;\\ E(s_{a}) & =\;\beta^{1/2}\frac{\Gamma (\alpha +0.5)}{\Gamma (\alpha )} \end{array}$$

Where *α* and *β* are computed as described in equations (C1), (C2) and (C5). The same equation can be applied to the computation of $E(s_{a_{m}})$. Therefore, we can calculate the proportion of standard deviation lost as:

$$PropSD=1-\frac{{\hat{\sigma }}_{a_{m}}}{{\hat{\sigma }}_{a}}\;=1-\frac{E(s_{a_{m}})}{E(s_{a})}$$

It is important to note that both $E(s_{a})$ and $E(s_{a_{m}})$ depend solely on $G\sigma_{a^{*}}^{2}$ and $G_{m}\sigma_{a^{*}}^{2}$. If the *G* matrix is multiplied by a scalar, it holds that $E(s_{a})$ gets multiplied by the square root of the same scalar. Therefore:

$$\begin{array}{rl} E(s_{a}\;|\;G\sigma_{a^{*}}^{2}) & =\sigma_{a^{*}}E(s_{a}|G)\\ E(s_{a_{m}}\;|\;G_{m}\sigma_{a^{*}}^{2}) & =\sigma_{a^{*}}E(s_{a_{m}}\;|\;G_{m})\\ PropSD & =1-\frac{{\hat{\sigma }}_{a_{m}}}{{\hat{\sigma }}_{a}}\;=1-\frac{\sigma_{a^{*}}E(s_{a_{m}}\;|\;G_{m})}{\sigma_{a^{*}}E(s_{a}\;|\;G)}\;=1-\frac{E(s_{a_{m}}\;|\;G_{m})}{E(s_{a}\;|\;G)} \end{array}$$

As a result, the REML estimation of $\sigma_{a^{*}}$ is not required for the computation of *PropSD*, only *G* and $G_{m}$ are needed.

## Appendix D. Inbreeding rate

Genetic diversity is often conceptualized in terms of inbreeding (*F*) for genomic mating Kinghorn (2011), Akdemir and Isidro y Sánchez (2016), Gorjanc and Hickey (2018) and Endelman (2025). However, while inbreeding can be computed accurately from a numerator relationship matrix (*A*) if a good pedigree is available (which is rare), it presents some error when computed from genomic relationships Wang (2014) and Endelman (2024). The likely reasons behind this are that: i) the *G* matrix captures identity by state, not only identity by descent, and ii) the scaling of the *G* matrix is often incorrect, as the VanRaden method relies on very simplistic and unrealistic assumptions Fernández-González and Isidro y Sánchez (2025). Thus, we recommend using *PropSD* instead. However, if desired, MateR also supports controlling diversity with the inbreeding rate (ΔF). ΔF is computed as Woolliams et al. (2015) and Endelman (2025):

$$\Delta F=\;\frac{F_{1}-F_{0}}{1-F_{0}}$$

Where $F_{0}$ is the inbreeding value in the parental pool and $F_{1}$ is the inbreeding value in the selected crosses. We can compute $F_{0}$ and $F_{1}$ as the average inbreeding of all genotypes in the corresponding generation. Inbreeding is often computed as the diagonal of the relationship matrix minus one, but this can be very problematic in some scenarios. For instance, for double haploids, all diagonal elements in *A* are always 2. Therefore, the average inbreeding for any population would be 1 regardless of how related the genotypes are to each other, i.e. we are not capturing group coancestry. Therefore, we need an alternative. According to Xu (2022), the inbreeding of an individual is equal to the average coancestry of its two parents, which itself is half their relationship in *A*. Therefore, $F_{1}$ can be computed as the mean of the off-diagonal elements of the *A* matrix for their parents divided by two:

$$F_{1}=\;\frac{1^{⊤}A_{m}\;1-\mathrm{tr}(A_{m})}{2n_{m}(n_{m}-1)}$$

Where $A_{m}$ is the numerator relationship matrix for the parents of the mating plan, with each parent replicated as many times as the number of crosses in which it is involved. $n_{m}$ is the number of rows or columns in $A_{m}$.

For $F_{0}$ it seems that we would need the *A* matrix for the previous generation, which is often unavailable. However, we know that, for four parents a,b,c,d and two offspring *ab* and *cd* obtained by crossing the respective parents, the relationship between them is Akdemir and Isidro y Sánchez (2016):

$$A[ab,cd]=\;\frac{A[a,b]+A[c,d]}{2}$$

Thus, $F_{0}$ can be computed as:

$$F_{0}=\;\frac{1^{⊤}A_{p}\;1-\mathrm{tr}(A_{p})}{2n_{p}(n_{p}-1)}$$

Where $A_{p}$ is the relationship matrix for the parental pool and $n_{p}$ is the number of parents.

If the *G* matrix is used instead, we can simply compute inbreeding as:

$$\begin{array}{rl} F_{1} & =\;\frac{1^{⊤}G_{m}\;1-\mathrm{tr}(G_{m})}{2n_{m}(n_{m}-1)}\\ F_{0} & =\;\frac{1^{⊤}G_{p}\;1-\mathrm{tr}(G_{p})}{2n_{p}(n_{p}-1)} \end{array}$$

It is very important to use the VanRaden VanRaden (2008) relationship matrix in this context, as it provides a scaling of *G* that makes it analogous to *A*. Furthermore, this method of computing inbreeding is the same as the third method in Morales-González et al. (2020). However, as explained earlier, using the *G* matrix will likely incur some errors due to improper scaling and contamination from identity by state.

## Appendix E. Standard error of the mean and selection intensity

To assess the diversity of a mating plan, we treat $P_{m}$ as a sample from the entire parental population, which is assumed to have mean-centered additive values (i.e. mean zero). Using ${\bar{P}}_{m}$ to denote the average additive value of the selected parents, we estimate the standard error of the mean (SEM) using the following expression, which accounts for correlations among samples Fernández-González and Isidro y Sánchez (2025):

$$SEM=\;sd({\bar{P}}_{m})=\frac{{\left(1^{⊤}G_{m}\sigma_{a^{*}}^{2}1\right)}^{1/2}}{n}$$

where $G_{m}$ is the genomic relationship matrix of the parents selected for the mating plan, 1 is a vector of ones, and *n* is the number of rows (or columns) in $G_{m}$. SEM can be related to selection intensity to make it more interpretable. ${\bar{P}}_{m}∼N(\mu =0,sd=SEM)$ has a zero mean because the sample mean is an unbiased estimator. We scale ${\bar{P}}_{m}$ to standard deviation units by:

$${\bar{P}}_{m}^{*}=\frac{{\bar{P}}_{m}}{\sigma_{a^{*}}}∼N\;\left(0,sd=\frac{{\left(1^{⊤}G_{m}1\right)}^{1/2}}{n}\right)$$

We assume that the overall parental average (*μ*) is zero due to mean-centered BLUPs. Therefore, it holds that ${\bar{P}}_{m}={\bar{P}}_{m}-\mu$, which can be interpreted as a selection differential. Similarly, ${\bar{P}}_{m}^{*}=\frac{{\bar{P}}_{m}}{\sigma_{a}}=\frac{{\bar{P}}_{m}-\mu }{\sigma_{a}}$ can be viewed as a selection intensity. Therefore, we can directly compare ${\bar{P}}_{m}^{*}$ with the actual selection intensity of the selected parents ($i_{realized}$), which can be calculated using the genomic estimated additive values of the parental genotypes. We are interested in the probability that the scaled average of the selected parents exceeds $i_{realized}$:

$$\text{Pr}\;\left({\bar{P}}_{m}^{*}>i_{realized}\;|\;G_{m}\right)=\alpha$$

This can be rearranged as:

$$Q(1-\alpha )\cdot \frac{{\left(1^{⊤}G_{m}1\right)}^{1/2}}{n}\;=i_{realized}$$

where Q(x) is the quantile function of the standard normal distribution. This allows us to compute *α*, which is the likelihood of a solution with a higher gain than the current mating plan while maintaining similar diversity.

MateR allows controlling diversity loss using SEM as a metric directly. It always computes *α* internally for any optimal mating plan, which allows evaluating the goodness of the optimization. The lower the value of *α*, the less likely it is that a solution better than the current one exists. Thus, low *α* values indicate good optimization quality.

It is important to note that *α* values are calculated based only on the additive genomic relationships. Thus, they are unable to capture dominance, and they become meaningless in the presence of heterosis.
