# Supplementary material for: MateR: a novel genomic mating framework
Source: Genetics. 2026 Jan 19;232(4):iyag013. doi: 10.1093/genetics/iyag013 (PMC13050210; doi:10.1093/genetics/iyag013)
Supplement: iyag013_Supplementary_Data [file iyag013_supplementary_data.zip › Supplementary_Material_Legends_GENETICS-2025-308633.docx]

**Supplementary File 1:** R script providing reproducible counterexamples showing that Falconer’s equation for computing the family mean does not hold across the tested scenarios (several F1 crosses under explicit dominance), with detailed comments and expected outputs.

**Supplementary File 2:** Excel workbook offering a feature-by-feature comparison of genomic mating software, including objective functions, parameterizations (additive/dominance), generation scope, polyploid support, input requirements (markers vs. phased haplotypes), and optimization capabilities.
